# Assessing predictors of contraceptive use and demand for family planning services in underserved areas of Punjab province in Pakistan: results of a cross-sectional baseline survey

**DOI:** 10.1186/s12978-015-0016-9

**Published:** 2015-03-28

**Authors:** Syed Khurram Azmat, Moazzam Ali, Muhammad Ishaque, Ghulam Mustafa, Waqas Hameed, Omar Farooq Khan, Ghazunfer Abbas, Marleen Temmerman, Erik Munroe

**Affiliations:** Department of Uro-gynecology, University of Ghent, Ghent, Belgium; Department of Reproductive Health and Research, World Health Organization, Geneva, Switzerland; Research, Monitoring & Evaluation Department, Marie Stopes Society, Karachi, Pakistan; Research, Monitoring and Evaluation Department, Marie Stopes International, London, United Kingdom

## Abstract

**Background:**

Although Pakistan was one of the first countries in Asia to launch national family planning programs, current modern contraceptive use stands at only 26% with a method mix skewed toward short-acting and permanent methods. As part of a multiyear operational research study, a baseline survey was conducted to understand the predictors of contraceptive use and demand for family planning services in underserved areas of Punjab province in Pakistan. This paper presents the baseline survey results; the outcomes of the intervention will be presented in a separate paper after the study has been completed.

**Method:**

A cross-sectional baseline household survey was conducted with randomly selected 3,998 married women of reproductive age (MWRA) in the Chakwal, Mianwali, and Bhakkar districts of Punjab. The data were analyzed on SPSS 17.0 using simple descriptive and logistic regression.

**Results:**

Most of the women had low socio-economic status and were younger than 30 years of age. Four-fifths of the women consulted private sector health facilities for reproductive health services; proximity, availability of services, and good reputation of the provider were the main predicators for choosing the facilities. Husbands were reported as the key decision maker regarding health-seeking and family planning uptake. Overall, the current contraceptive use ranged from 17% to 21% across the districts: condoms and female sterilization were widely used methods. Woman’s age, husband’s education, wealth quintiles, spousal communication, location of last delivery, and favorable attitude toward contraception have an association with current contraceptive use. Unmet need for contraception was 40.6%, 36.6%, and 31.9% in Chakwal, Mianwali, and Bhakkar, respectively. Notably, more than one fifth of the women across the districts expressed willingness to use quality, affordable long-term family planning services in the future.

**Conclusion:**

The baseline results highlight the need for quality, affordable long-term family planning services close to women’s homes. Furthermore, targeted community mobilization and behavior change efforts can lead to increased awareness, acceptability, and use of family planning and birth spacing services.

## Background

Family planning (FP) is one of the most “health-promoting” and cost-effective activities in public health promotion and has the potential to avert approximately 30% of maternal and 10% of child deaths [1]. Thus, FP contributes to achieving the Millennium Development Goals (MDGs) through healthier birth spacing and by reducing mortality and morbidity associated with pregnancy [2]. In 1950, Pakistan had a population of 37 million and was the world’s 13^th^ largest country as measured by population; however, in 2013, Pakistan had become the sixth largest country with 191 million people [3]. The first government-supported family planning program was started in the 1960s, but over a 50-year period, priorities changed as the program evolved. Failure to effectively manage the fertility rate and rapid population growth had adverse effects on development indicators such as education, poverty, and life expectancy, particularly for maternal and child health [4].

In Pakistan, approximately 35% (more than 8 million) of married women of reproductive age (MWRA) practice some form of family planning, and of them, around 26% (6 million women) use a modern method [5]. Among modern methods, female sterilization/tubal ligation is the most common method at around 45% of the modern method mix, but it is chosen late, often after 31.5 years of age and usually after four or more children [5]. Second, short-term methods such as condoms accounted for around 23% of the method mix, with the remainder divided between the pill, injection, and long-term method (LTM) for contraception, i.e., an intrauterine contraceptive device (IUCD): 8%, 7%, and 17%, respectively [5]. A Pakistani woman does not enjoy autonomy of decision making regarding her own reproductive health and family planning needs [6]. Moreover, religious opposition and misinterpretation of family planning impede the adoption of contraceptives, even among those who desperately want to space their children [7].

Pakistan is a signatory to the International Conference on Population and Development (ICPD). To achieve the country’s commitment to the global MDGs to lower the fertility rate, slow the rapid population growth, and decrease maternal, neonatal, and child morbidity and mortality, the government of Pakistan pledged to increase the contraceptive prevalence rate to 55% by 2015 [8]. However, with the prevailing scenario, it is improbable that this goal will be achieved, although the government of Pakistan is still the major FP provider in Pakistan (47%) while 23% of the need is fulfilled by the private sector [5]. Importantly, 53% of the LTM is supplied by the public sector, but their contribution in the modern contraceptive prevalence rate (CPR) is only 2.3% [5]. Thus, concentrated efforts are required to utilize all available channels in the public, private, and nongovernmental organization (NGO) sectors to address the low and very slowly progressing CPR and high unmet need for family planning in Pakistan since only with public and private sector collaboration can this challenging goal be achieved.

In the present scenario, franchised health establishments are becoming popular worldwide in attracting reproductive health clients [9]. The Marie Stopes Society (MSS), a local non-governmental organization, therefore, originally piloted its own version of social franchise intervention as Suraj (i.e., in English, Sun, a brand name provided to the clinics of the trained franchise providers of MSS) in 2008 to improve the reproductive health of women living in rural communities and to test the intervention’s feasibility [10]. The earlier findings documented increased awareness and modern contraceptive use especially of IUCDs in targeted rural poor communities in a few districts in Punjab and Sindh provinces [10,11]. Thus, with slight modifications of the original MSS Suraj social franchise model [10-12], the MSS implemented a new operational research project, with a quasi-experimental study design. Based on a similar social franchising model with a demand-side financing approach, the MSS built a partnership with local health care providers in underserved rural areas of Pakistan to produce robust results within a minimum period of time to increase the targeted CPR among the poorest [12].

As part of this multiyear operational research study, a baseline survey was conducted in May 2012 to understand the predicators of contraceptive use and demand for family planning services in underserved areas of Punjab province in Pakistan. This paper presents the survey results; the outcomes of the intervention will be presented in a separate paper after the study has been completed. It is anticipated that the baseline findings will inform the operations research project implementation and programmatic decision making to ensure that the project is on track and adequately meets the contextualized supply and demand needs in the targeted areas. Moreover, the baseline survey will serve as a benchmark to assess the effect of the intervention. The end-line survey of this project will be conducted after the 24-month intervention has been completed to measure change in key project indicators as a result of the intervention.

## Methodology

### Study design

This is a quasi-experimental study with a control arm. Data collection methods include population-based household surveys (baseline and end-line).

### Study settings

The survey was conducted in two intervention districts (Chakwal and Mianwali) and the control district (Bhakkar), where the control district was selected based on its proximity and comparability to demographic and service delivery indicators of the intervention areas. The control district is at a sufficient distance from the intervention districts to any contain spillover effect of the intervention.

### Sampling

The multi-stage sampling technique was used to select respondents. At the first stage, the districts and catchment area of the providers were purposively selected. The catchment area for the providers spread over 3–4 km radius and were at a sufficient distance from one another. After demarcation of the catchment area, all households within the catchment area of each service provider were allotted a unique study number in order to select the households for the baseline survey. Using computer software, we randomly selected a sample of households within the catchment of each service provider.

### Sample size

The target population for the baseline survey included MWRA (15–49 years). Overall, a total of 3,998 MWRA were interviewed: 694 in Chakwal, 719 in Mianwali, and 2,585 in Bhakkar. One MWRA was interviewed in a household. If more than one MWRA was found within a household, then the youngest MWRA was interviewed.

### Tool for baseline survey

The survey questionnaire was adapted from the 2006–07 Pakistan Demographic Health Survey (PDHS) instruments. The questionnaire had two components: the demographic and socio-economic characteristics of women and one that explored fertility choices, contraceptive knowledge, and practices and aspects of reproductive health.

### Outcome variable

All respondents were asked about the current use of contraceptive methods. This outcome variable (current use of contraceptives) was asked similarly on the 2006–2007 Pakistan Demographic Health Survey. If a woman reported current use of any contraceptive method, the response was coded 1 and 0 if the response was negative.

### Independent variables

Many socio-demographic and other factors may influence the decision of MWRA to use contraceptives. We included all socio-demographic and other relevant variables in the model to determine the association between the dependent and independent variables. The list of variables used for the analysis include age of the woman and her husband; woman’s age at marriage; education and occupation of the woman and her husband; number of the living children the couple have; wealth quintiles; knowledge about and past and current use of contraceptives; attitudes toward family planning and birth spacing; decision-making dynamics regarding health seeking, family planning, pregnancy, and delivery; and spousal communication regarding family planning and birth spacing.

### Statistical analysis

Data were analyzed using SPSS 17.0. Descriptive statistics, frequencies, and proportion were run based on the respondents’ socio-demographic characteristics, reproductive health care profile, decision making, contraceptive knowledge, attitude, and practice. The mean, median, and standard deviation were calculated for the continuous variables and proportions for the categorical variables. Univariate logistic regression analysis was applied to determine the association between the outcome variable (current contraceptive use) and risk factors (couple’s age and education, age at marriage, number of children, decision making, place of delivery, and wealth quintile). The adjusted odds ratio (AOR) and the 95% confidence interval were also calculated by applying multivariable logistic regression analysis. Variables that were statistically significant or meaningful were entered in the multivariable logistic regression model.

### Ethical consideration

The study protocol, informed consent form, and baseline household survey questionnaire were reviewed and approved by the Ethics Review Committee (ERC) of the National Bioethics Committee (NBC) of Pakistan [12].

## Results

### Socio-demographic characteristics of MWRA

The mean age of the respondents was 31 ± 6.9 years, and the mean age at the time of marriage was 20 ± 3.5 years. Illiteracy among MWRA was higher in Mianwali and Bhakkar (60%) compared to Chakwal (32%). Mean household income was PKR11, 111 ± 9,695 (US$104 ± 90.4) per month (refer to Table 1).

**Table 1:** **Socio-demographic characteristics of MWRA**

**Characteristics**	**Chakwal %**	**Mianwali %**	**Bhakkar %**
**Age of women**			
≤24	15.3	16.3	15.5
>24 to 35	43.2	45.3	53.3
>35	41.5	38.4	31.2
Average ± SD	32.4 ± 7.4	31.2 ± 7.1	30.3 ± 6.3
**Age of women at marriage**			
≤20	67.4	65.0	65.7
>20 to 25	25.9	26.6	29.8
>25	6.6	8.5	4.4
Average ± SD	19.8 ± 3.7	20.2 ± 3.8	19.9 ± 3.3
**Age of husband**			
<30	19.7	22.4	21.9
30 to <40	34.6	37.7	46.8
40+	45.7	39.9	31.4
Average ± SD	37.1 ± 8.7	37.1 ± 8.6	34.9 ± 7.5
**Women’s education**			
Illiterate	32.6	60.6	63.4
Literate	67.4	39.4	36.6
**Husband’s education**			
Illiterate	11.2	23.8	38.1
Literate	88.8	76.2	61.9
**Number of living children**			
No children	3.8	6.6	5.7
1 child	18.2	18.7	17.5
2-3 children	39.9	36.4	38.4
4 or more children	38.2	38.4	38.4
**Wealth quintile**			
Poorest	5.9	14.9	25.2
Poor	13.3	21.2	21.4
Middle	15.5	18.1	21.7
Rich	19.6	23.6	19.1
Richest	45.7	22.2	12.5

### Health care seeking and decision making

**Figure 1:**
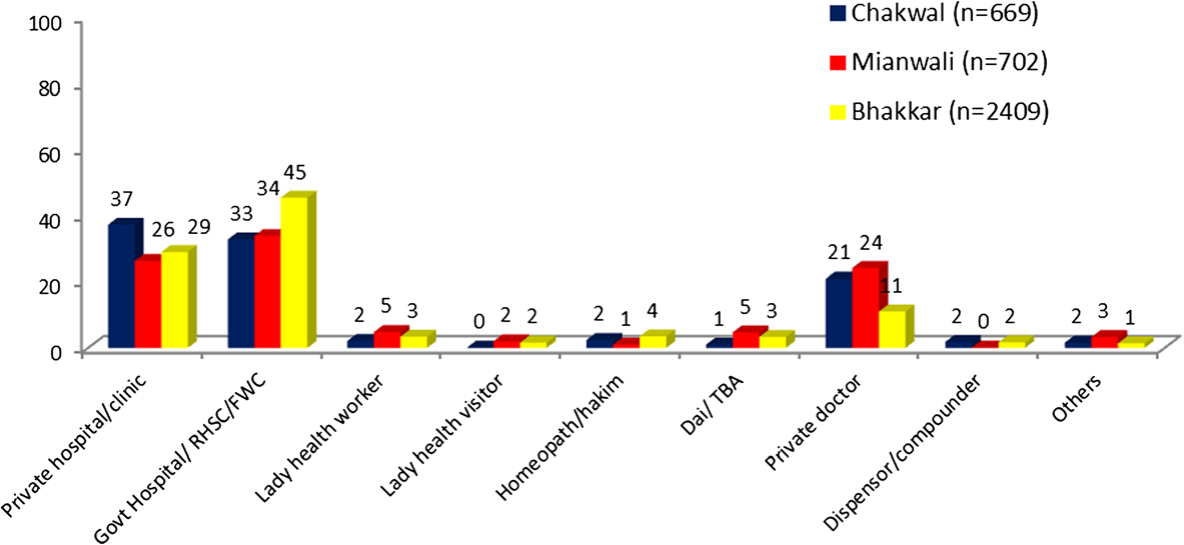
**Type of facility and provider for seeking RH care.**

Most of the MWRA in Bhakkar (45%) and Mianwali (34%) consulted public sector health facilities such as reproductive health service centers (RHSCs) and family welfare clinics (FWCs) for reproductive health (RH) care (refer to Figure 1). However, in Chakwal the majority visited private sector hospitals/clinics. In addition, a significant number of MWRA across the districts also visited private sector practitioners/doctors for RH care. The MWRA were also asked at the baseline to describe the attitude of the RH providers to their patients. Most of the MWRA across the three districts described the RH providers’ attitudes as either “somewhat helpful” (47%) or “very helpful” (42%).

**Figure 2:**
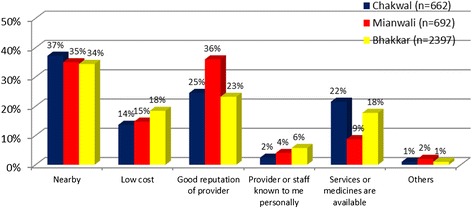
**Reasons for selecting a particular RH facility or a provider.**

An important indicator of quality service delivery is the “availability of medicines and contraceptives supplies” in a district. All three districts had majority of the medicines and supplies usually available (Chakwal: 86%; Mianwali: 92%; Bhakkar: 86%). In addition, the most of the MWRA in all three districts on average went alone to the facility/provider for RH services (66%). Women in Bhakkar were most likely to be more autonomous in terms of social mobility while seeking RH care (79%) followed by Mianwali (65%) and Chakwal (54%), respectively. A small number of MWRA in these districts reported they were accompanied by their husband (14%), brother (12%), relative/friend (6%), or mother-in-law (2%). Likewise, the common reasons for selecting a health facility or provider included proximity, good reputation of provider, and availability of services followed by the cost of services (refer to Figure 2).

**Figure 3:**
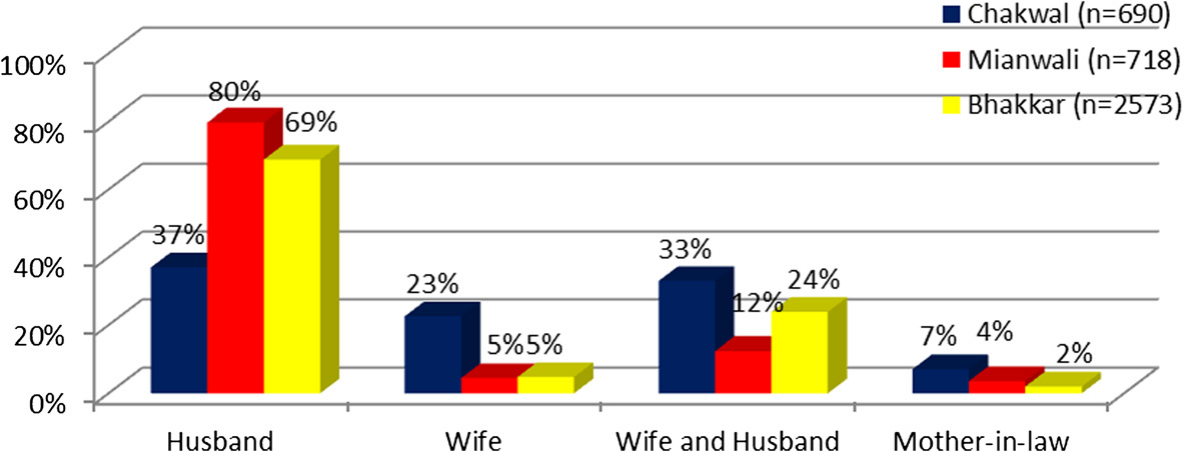
**Who makes the decisions in your household about RH care-seeking?**

Importantly, the median cost of RH services per recent visit reported by the respondents was PKR500 (US$4.65) and PKR800 (US$7.45) for Chakwal and Mianwali, respectively, but PKR300 (US$2.8) for Bhakkar. Meanwhile, the decisions in households regarding health care seeking were usually made by husbands for 80% of the respondents in Mianwali and 69% in Bhakkar (refer to Figure 3). In Chakwal, about 37% reported their husbands were the decision maker, 23% reported the wife was the decision maker, and 33% reported that the wife and the husband decided together about health care seeking, perhaps reflecting the slightly higher level of education and better economic situation in the district. Health care–seeking decisions about sick children were predominantly made by the husband with only occasional input from the wife. However, this pattern was less strong in Chakwal.

We also asked the MWRA about different methods that they had heard of and the sources of information. Knowledge about family planning and various modern contraceptive methods was considered universal in Pakistan [5]; however, the data indicate that this is not the case. Some of the results are shown in Table 2.

**Table 2:** **Knowledge about modern contraceptive methods**

**Method**	**Chakwal**	**Mianwali**	**Bhakkar**
	**N = 692**	**N = 718**	**N = 2583**
Pill	47%	55%	45%
IUCD	38%	38%	32%
Injections	42%	50%	41%
Condom	39%	34%	32%
Periodic abstinence	26%	17%	15%
Withdrawal	28%	14%	11%
Female sterilization	31%	22%	21%
Male sterilization	17%	11%	13%
Implants	16%	10%	9%

### Knowledge and practices of family planning contraception

In all three districts, the main source of information on abstinence, withdrawal, condoms, pills, and injections was a lady health worker (LHW). For IUCD, implants, and sterilization, respondents mentioned a lady health visitor (LHV) or a doctor as their primary source of information. The current contraceptive use across the districts was lower compared to the national CPR of 35% as reported in the latest Demographic Health Survey (DHS) [5]. Overall, the current contraceptive use in the surveyed districts ranged from 17% in Mianwali to 18% in Bhakkar and 21% in Chakwal. Interestingly, much lower use of traditional methods across the districts was reported than the national average. The main predictors of contraceptive use in all three districts were the age of the woman, whether she had achieved ideal family size, affluence, and access to an LHW or counseling; the last was the most powerful influence (refer to Table 3). Condoms were the most common method in use; around a third of the respondents used this method. The next most common method was female sterilization.

**Table 3:** **Unadjusted and adjusted odds ratios of current contraceptive use by socio-demographic characteristics of participants**

**Characteristics**	**N**	**n (%)**	**OR (95% CI)**	**AOR (95% CI)**
**Age of women**				
≤24	724	78 (10.8)	1	
>24 to 35	2,117	393 (18.6)	1.88 (1.45-2.44)***	
>35	1,591	297 (18.7)	1.90 (1.45-2.48)***	
**Age of women at time of marriage**				
>25	250	26 (10.4)	1	1
>20 to 25	1,141	159 (13.9)	1.42 (0.91-2.20)	1.01 (0.60-1.74)
≤20	2,039	583 (19.2)	2.07 (1.37-3.15)***	1.18 (0.70-1.98)
**Age of husband**				
<30	1,002	129 (12.9)	1	1
30 to <40	1,775	306 (17.2)	1.41 (1.13-1.77)***	0.58 (0.43-0.79)***
40+	1,654	333 (20.1)	1.70 (1.37-2.13)***	0.53 (0.38-0.73)***
**Women education**				
Illiterate	2,528	354 (14.0)	1	1
Literate	1,903	413 (21.7)	1.70 (1.45-1.99)***	1.22 (0.9-1.54)
**Husband education**				
Illiterate	1,140	195 (17.1)	1	1
Literate	3,291	573 (17.4)	1.02 (0.85-1.22)	0.67 (0.52-0.87)**
**Number of living children**				
0-1 child	1,017	76 (7.5)	1	1
2-3 children	1,478	305 (20.6)	3.21 (2.50-4.19)***	3.19 (2.28-4.47)***
4 or more children	1,586	387 (24.4)	3.98 (3.07-5.17)***	6.10 (4.20-8.86)***
**Wealth quintile**				
Poorest	852	97 (11.4)	1	1
Poor	919	138 (15.0)	1.38 (1.05-1.82)*	0.98 (0.69-1.39)
Middle	814	148 (18.2)	1.73 (1.31-2.29)***	1.54 (1.08-2.20)*
Rich	859	173 (20.1)	1.97 (1.50-2.58)***	1.72 (1.20-2.47)**
Richest	963	204 (21.2)	2.09 (1.61-2.72)***	1.41 (0.97-2.05)
**Decision making regarding pregnancy**				
Husband decides	3,088	521 (16.9)	1	1
Mother-in-law decides	225	38 (16.9)	0.99 (0.69-1.42)	1.14 (0.73-1.80)
Respondent (women decide)	298	60 (20.1)	1.23 (0.92-1.66)	1.17 (0.82-1.69)
Both (husband and wife) decide	632	122 (19.3)	1.18 (0.94-1.46)	1.04 (0.80-1.37)
**Spousal communication regarding family planning and birth spacing**				
No	1,765	71 (4.0)	1	1
Yes	2,655	697 (26.3)	8.48 (6.59-10.91)***	5.29 (4.22-6.65)***
**Spouses’ agreement on couple using contraceptive method is acceptable**				
Disagree by both	1,084	50 (4.6)	1	1
Agree by any (husband or wife)	408	65 (15.9)	3.94 (2.70-5.81)***	1.80 (1.60-2.80)**
Agree by both	2,228	607 (27.2)	7.82 (5.80-10.55)***	4.53 (3.26-6.30)***
**Place of last child delivered**				
Home	2,132	367 (17.2)	1	1
Maternity Home/Private clinic	283	65 (23.0)	1.43 (1.06-1.93)*	1.30 (0.89-1.89)
Hospital	1,472	333 (22.6)	1.40 (1.19-1.66)***	1.53 (1.24-1.88)***

P-value: *p < 0.05, **p < 0.01, *** p < 0.001.

Although the most common methods in current use included condoms and female sterilization, the use of female sterilization was high in Chakwal (5.3%) compared with Mianwali (2.8%) and Bhakkar (2.7%). The average current method mix for short-acting methods such as pills, injections, and condoms was the same as the long-acting method of contraception; for example, IUCD use was highest in Mianwali (3.3%) followed by 2.3% in Chakwal and 1.85% in Bhakkar (refer to Table 4).

**Table 4:** **Current contraceptive use and method-wise utilization trends**

	**Chakwal**	**Mianwali**	**Bhakkar**
	**N = 694**	**N = 719**	**N = 2585**
**Overall current contraceptive use**			
**Current contraceptive use**	**21%**	**17%**	**18%**
**Method-wise current contraceptive use**			
Pill	1.9%	2.2%	2.1%
IUCD	2.3%	3.3%	1.8%
Injections	2.9%	2.6%	2.4%
Condom	6.9%	5.6%	6.5%
Periodic Abstinence	0.4%	0.1%	1.5%
Withdrawal	1.3%	0.4%	0.4%
Female sterilization	5.3%	2.8%	2.7%
Male sterilization	0.0%	0.3%	0.1%

Moreover, in all three districts, more than half of the MWRA reported that that use of family planning methods to avoid pregnancy was acceptable while around 42% considered that use of modern contraception was acceptable in Islam (refer to Table 5).

**Table 5:** **Use of contraceptive method approved by women and their husbands**

**Indicators**	**Chakwal**	**Mianwali**	**Bhakkar**	**Overall**
	**N = 694**	**N = 719**	**N = 2585**	**N = 3998**
Mutually approved that couple using contraceptive method is acceptable	52.2%	53.5%	51.5%	52.0%
Mutually approved that couple using contraceptive method is acceptable in their religion	37.3%	45.3%	41.7%	41.6%

**Figure 4:**
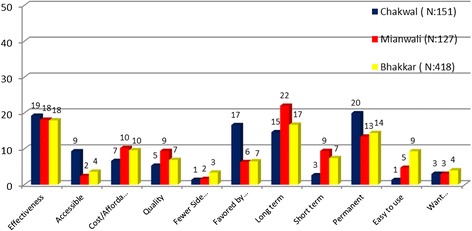
**Reasons for choosing a specific method.**

The most common reasons for choosing the contraceptive method currently in use, as reported by the MWRA, included perceived effectiveness of the contraceptive method followed by long-term effectiveness, permanent method, and affordability. Other reasons cited for choosing the current contraceptive method included quality and ease of access and use (refer to Figure 4). Husband’s or mother-in-law’s approval was also cited as an important reason for selecting the particular method currently in use.

The most common source for acquiring the current contraceptive method in use across the districts was either a government health facility or an LHW. Thus, public sector sources accounted for nearly half of the cases. The other common sources were private sector hospital/clinics and self-procurement from a private sector store or friends/relatives. Individual private sector practitioners/doctors were also cited as sources of family planning services (refer to Table 6).

**Table 6:** **Source of current contraceptive method**

**Source of current contraceptive method**	**Chakwal**	**Mianwali**	**Bhakkar**
	**N = 133**	**N = 118**	**N = 397**
Government hospital/RHSC	29.3%	27.1%	28.2%
Lady health worker	22.6%	22.9%	24.4%
Private hospital/clinic	8.3%	11.9%	14.6%
Shop/friends/relatives/husbands	17.3%	11.0%	10.6%
Lady health visitor	3.0%	2.5%	7.6%
Private doctor	6.0%	6.8%	3.8%
Pharmacy/dispenser/compounder	1.5%	5.9%	4.5%
Family welfare center	2.3%	5.9%	1.3%
RHC/BHU/MCH	0.0%	0.8%	0.8%
Others (push cart/Dai/TBA/mobile service camp)	9.8%	5.0%	4.3%

In addition, the median distance to the nearest health facility or provider for FP services was 1 km in Chakwal, 2 km in Mianwali, and 3 km in Bhakkar.

As mentioned in Table 7, the cost associated with use of services also varied in all three districts; the median cost of the FP method was PKR85 (US$0.80) in Chakwal, PKR125 (US$1.17) in Mianwali, and PKR200 (US$1.87) in Bhakkar.

**Table 7:** **Cost of FP services**

**Median cost of FP method**	**Chakwal**	**Mianwali**	**Bhakkar**
	**PK Rupees (US$)**	**PK Rupees (US$)**	**PK Rupees (US$)**
Condoms (12 units)	50 (US$0.47)	150 (US$1.40)	130 (US$1.21)
Pills	130 (US$1.21)	30 (US$0.28)	200 (US$1.87)
Injections	55 (US$0.51)	100 (US$0.93)	95 (US$0.89)
IUCD	60 (US$0.56)	200 (US$1.87)	400 (US$3.74)
Female sterilization	–	1265 (US$11.82)	2000 (US$18.69)
Male sterilization	–	9 (US$0.08)	–

Regarding the LHW’s visit to households for FP information/counseling or short-term methods, 69% of the MWRA in Mianwali, 65% in Bhakkar, and 51% in Chakwal reportedly received a visit in the 1 month before the baseline survey. Around 10% of the MWRA reported having seen a family planning message on television during the same period. Among those who saw the advertisement, the majority (87%) mentioned that it conveyed the message of increasing birth spacing, and almost a similar percentage of MWRA (85%) found the message effective in spreading awareness and benefits of birth spacing.

### Fertility preference, unmet need for contraception, and willingness to use contraception in the future

Around half of the MWRA across the three districts stated that they wanted to have another child. The most common reason was the in-laws’ desire for a male child. In all three districts, for the newly married couples, more than 40% would prefer having their first child born within first year, and that was mainly due to family pressure.

The baseline results reveal high need for contraception at 40.6%, 36.6%, 31.9% in Chakwal, Mianwali, and Bhakkar, respectively (refer to Table 8). Notably, 31.3%, 22.6%, and 20.7% of the women in Mianwali, Chakwal, and Bhakkar, respectively, expressed their willingness to use family planning services in the future. Long-term effectiveness (Chakwal 34.2%, Mianwali 20.7%, Bhakkar 33.5%), quality (Chakwal 29.9%, Mianwali 18.4%, Bhakkar 17.4%), and affordability (Chakwal 15.4%, Mianwali 22.4%, Bhakkar 21.8%) were cited as important factors that would influence MWRA choice of contraception in the future. The willingness to pay for family planning was highest in Chakwal where up to 75% of the women were willing to pay, if they were offered quality services, compared to Mianwali (60%) and Bhakkar (53%).

**Table 8:** **Assessing need and willingness and reasons to use contraception in the future**

**Indicators**	**Chakwal %**	**Mianwali %**	**Bhakkar %**
**Assessing need for contraception**	40.6	36.6	31.9%
**Willing to use contraception in the future e**	22.6	31.3	20.7
**Method preference (among those who will use a contraceptive method in the future)**			
Condoms	24.4	10.8	21.1
Pills	17.9	19.5	16.1
Injections	14.6	16.2	17.7
Implant	6.5	2.7	1.6
IUCD	12.2	8.1	9.8
Female sterilization	13.0	20.5	12.3
Male sterilization	0.0	0.0	0.0
Periodic abstinence	0.8	0.0	2.5
Withdrawal	2.4	0.0	1.1
Don’t know/whatever suggested by husband	8.2	22.1	17.7
**Reason for choosing a specific contraceptive method in the future**			
Cost/affordability	15.4	22.4	21.8
Quality	29.9	18.4	17.4
Long term	34.2	20.7	33.5
Short term	4.3	2.9	6.9
Permanent	12.0	17.3	9.9
Better for health	1.7	1.1	5.7
Easy access	0.9	0.0	0.0
Others (suggested by husband/for spacing)	0.0	2.3	0.2
Don’t know/	1.7	14.9	4.5

## Discussion

Women’s social position and health care utilization patterns call for establishing a model in women have the permission, ability, and trust to seek and obtain the services of their choice [13]. More than half of the women reported they received reproductive health services from public sector outlets, which reflects the widespread presence of public sector health networks. In addition, a significant proportion of women also reported they received FP/RH services from private sector sources. The increasing use of the private sector for RH health care reflects the national trend and women’s concern for quality of care associated with private sector health services [5,14]. To address the cost incurred in such consultations in the private sector, vouchers are a tested model for providing a safety net to the poor to use the quality services needed [10].

Many women described they were able to go RH services alone, which is much higher than what has been described in more conservative communities, for example, in some of the southern and northern regions of the country [15,16]. Another striking result of our study is the reason for choosing a specific provider: the reputation of the provider and the distance to the facility. Most women in Pakistan find it easy to see a health provider, if he or she is in the vicinity and is already known in the community [17]. Despite the autonomy that women enjoyed in terms of mobility, decisions regarding various household affairs rest with the men or the head of the household, which is a very peculiar feature of patriarchal society [18].

The findings indicate that overall knowledge of particular methods such as male condoms is very low; for example, only around half of the women had even heard about any of the common modern methods. This is in contrast with national data that reported almost universal knowledge of contraceptive methods and male condoms as one of the two most popular modern contraceptive methods known and used in Pakistan [5]. This fact can be attributed to the lack of inter-spousal communication, which is almost negligible in these districts. The proposed franchising intervention has a strong component of couples counseling whenever a client approaches the FP provider for birth spacing consultation. Women who are aware and knowledgeable about the methods mentioned LHWs as their primary source for most methods. This is in agreement with findings for the LHW program in Pakistan [19]. Since only 60% of the women reportedly received an LHW visit during the 1 month before this baseline survey, the national program must strategize how the efficiency of the LHWs can be further increased: either by recruiting and deploying more workers or by strengthening the monitoring and supervision of the LHWs. Field health educators (FHEs) of the franchised FP/RH programs might also be helpful in filling the gaps in areas where it is difficult for LHWs to visit certain households more frequently.

The current contraceptive usage rates are around 20% in these districts, much lower than the 35% that was reported in the 2012–13 Pakistan Demographic and Health Survey [5]. Moreover, the unmet need for contraception as documented in this survey is much higher and almost twice the national average [5]. Most importantly, almost one in four MWRA currently not using any contraception expressed the desire to use contraception in the future. However, long-term effectiveness followed by quality and affordability were cited as the main reasons for choosing a specific contraceptive method in the future. Franchising private sector providers in the two intervention districts has the potential to improve access as well as choice of family planning services for MWRA. This model has been demonstrated to work in other countries where the government system has not worked to build trust and thus deliver quality family planning services [20]. The rates of use of traditional methods were much lower than the national average, which also reflects the lack of awareness of birth spacing methods and highlights the need for increasing awareness of the full range of family planning methods. Among modern methods, condoms were the most popular method, for the same reason often documented in national surveys as well as other studies, i.e., ease of access and ease of use [5,21]. Field health educators/workers and community mobilizers in family planning programs along with LHWs must reinforce messages about long-term methods, to meet the needs of couples who are looking for long-term child spacing. The baseline results also revealed a desire for more children, especially the desire for a son, as a dominant reason for currently not using contraception, which reflects the general trend [5] that women adopt contraception late in life when they have achieved their desired family size. The FP programs can also benefit from behavior change efforts aimed at promoting birth spacing during early years of marriage and to promote optimal birth intervals. Religion or more precisely religious interpretations by local clergy are also an important barrier to using modern FP methods. This issue must be tackled more broadly by engaging actively with local community leaders, social workers, village elders, and renowned religious scholars [22]. The key limitations of our findings are mostly related to generalizability since the data were collected through a cross-sectional survey from intervention sites in only three districts of Punjab province in Pakistan. Furthermore, the interviews were conducted only among women; therefore, the study did not capture in-depth information about men's perspectives on fertility or about their desire and intentions regarding contraceptive use.

## Conclusion

The baseline results emphasize the predicators of contraceptive use in rural Pakistan, which include proximity, availability of quality and affordable services, positive perceptions of family planning, spousal communication, and in-laws’ approval. Overall, the results highlight the need for quality, affordable long-term family planning services close to women’s homes. In addition, targeted community mobilization and behavior change efforts can address socio-cultural issues and misconceptions related to family planning and modern contraception and lead to increased awareness, acceptability, and use of modern contraception.
